# Optimizing Granulocyte Colony-Stimulating Factor Transcript for Enhanced Expression in *Escherichia coli*

**DOI:** 10.3389/fbioe.2021.630367

**Published:** 2021-03-09

**Authors:** Sonal Datta

**Affiliations:** Council of Scientific and Industrial Research, Institute of Microbial Technology, Chandigarh, India

**Keywords:** messenger RNA engineering, G-CSF, optimizing transcript for recombinant protein expression, stable secondary structures in mRNA, translation efficiency

## Abstract

The human granulocyte colony-stimulating factor (G-CSF) is a hematopoietic growth factor used to prevent and treat neutropenia. G-CSF stimulates the bone marrow to produce infection-fighting granulocytes. Food and Drug Administration of the United States approved G-CSF in 1991 and its PEGylated version in 2002 as a prophylactic and therapeutic measure against neutropenia. Recombinant human G-CSF is produced in surrogate host *Escherichia coli* and is PEGylated at N-terminal. Besides neutropenia, G-CSF is also used in bone marrow transplantation for the mobilization and maturation of peripheral blood stem cells. Considering the requirement of producing G-CSF therapeutic in large quantities, construct designing for high expression is critical for the biopharmaceutical and industrial application. Earlier studies have employed approaches such as codon optimization, use of strong promoters, employment of protein tags, secretion signals, optimization of protein folding, etc., for increasing expression and yield of therapeutic proteins. In this study, it was observed that mRNA transcribed from the native human cDNA of G-CSF and the codon-optimized variant leads to low protein expression in *E. coli*. To understand the underlying reasons, the mRNA secondary structure of the 5′ end of the G-CSF transcript was analyzed. This analysis revealed the presence of stable secondary structures at the 5′ end of the G-CSF transcript, arising from the native human gene and even from the codon-optimized sequence. These secondary structures were disrupted through translationally silent mutations within the first 24 nucleotides of the transcript without affecting the protein sequence. Interestingly, through this approach, the G-CSF protein expression was increased 60 folds as compared to native G-CSF construct. We believe that these findings create a roadmap for optimization of G-CSF transcript for enhanced expression in *E. coli* and could be employed to increase the expression of other therapeutic proteins.

## Introduction

Human granulocyte colony-stimulating factor (G-CSF) is a hematopoietic cytokine that plays a critical role in the stimulation, proliferation, mobilization, maturation and activation of granulocytes, including neutrophils (Anderlini et al., 1996; Welte et al., 1996). Neutrophils are one of the most abundant cell types amongst the leukocytes and thus play a critical role in the clearance of invading pathogens and modulate immune system homeostasis (Malech et al., 2014). A decrease in circulating neutrophils is called neutropenia, and it often leads to systemic infections and associated problems (Lyman and Kuderer, 2002). Due to its ability to induce proliferation and activation of neutrophils, G-CSF is clinically used to prevent and treat cancer chemotherapy-induced neutropenia (Crawford et al., 2004; Lyman, 2006). Over the years, G-CSF and its PEGylated forms have become the most valuable therapeutic proteins (Dale, 2002).

For clinical interventions, a recombinant form of G-CSF produced in *Escherichia coli* is utilized. The recombinant G-CSF differs from the native human G-CSF through the addition of N-terminal methionine for facilitating the expression in the surrogate host, and it lacks inherent glycosylation at Threonine-133 as observed in humans. However, the recombinant G-CSF possesses similar biological activity as that of the native G-CSF (Frampton et al., 1994). G-CSF is a 18.8 kDa protein consisting of one free cysteine and two disulfide bonds. Clinically used PEGylated form of G-CSF is also derived from recombinant G-CSF produced in *E. coli*. G-CSF’s recommended dosage regimen is 230 μg/m^2^/day for 2 weeks (Frampton et al., 1994), while 6 mg of PEGylated G-CSF is administered once per cycle of chemotherapy as a prophylactic and therapeutic measure against neutropenia (Rifkin et al., 2010). These dosages suggest that recombinant G-CSF needs to be produced in large quantities as a recombinant protein in *E. coli*. Given the requirement of large quantities of G-CSF therapeutic protein, construct designing for high expression of G-CSF is critical for managing production costs. Several approaches are employed to produce this protein in higher quantities, including codon optimization, use of strong promoters, employment of protein tags, secretion signals, etc. (Jin et al., 2011; Do et al., 2014; Zhou et al., 2016). In this study, I have employed mRNA transcript engineering for increasing the G-CSF expression levels in the *E. coli* expression system. This approach has resulted in a notable increase in the expression of functional G-CSF.

## Materials and Methods

### Materials

The details of all chemicals, molecular biology reagents, G-CSF standard, protein purification resins, columns, etc., were reported in Kumari et al. (2020). Protein concentrators were procured from Merck Millipore. RNA isolation and DNase treatment kits were procured from Qiagen. cDNA synthesis kit was procured from Bio-Rad Laboratories, Inc. Highest analytical grade reagents were used in the current study. AKTA Pure M (25M1) chromatographic system was utilized for protein purification, and analysis was performed using Unicorn 7.3 software. All cell culture reagents were from Gibco, Thermo Fisher Scientific.

### G-CSF Transcript Engineering to Increase the Protein Expression in *E. coli*

The human G-CSF cDNA, codon-optimized gene sequence and cloning details are as provided earlier (Kumari et al., 2020). Briefly, the native human G-CSF cDNA sequence was PCR amplified using forward primer SD13 and reverse primer SD14 and cloned in pET 23a. The *E. coli* codon-optimized G-CSF sequence was also cloned at *Nde*I/*Hin*dIII sites of the expression vector pET 23a (Novagen) using forward primer SD1 and reverse primer SD2 (primer sequences are provided in Table 1). Translationally silent mutations were incorporated in the codon-optimized gene sequence to engineer the G-CSF transcript’s 5′ region. The incorporation of translationally silent mutations was confirmed by DNA sequencing. The constructs were transformed in *E. coli* BL21 (DE3) from Novagen.

**TABLE 1:** Details of primers used in this study.

SD1	5′ ATGACGCCGCTGGGTCCG 3′
SD2	5′ CGGCTGTGCCAGGTGAC 3′
SD8	5′ ATG ACT CCA TTA GGT CCA GCA TCT AGC CTG CCG CAA 3′
SD11	5′ CTG CAA CCG ACG CAA GGT GCC ATG 3′
SD12	5′ GTG ACC CAG CAG GAC CAG TTC TTC CGG 3′
SD13	5′ ATG ACC CCC CTG GGC CCT 3′
SD14	5′ GGG CTG GGC AAG GTG GCG 3′

### Purification of G-CSF

The G-CSF constructs used in the study were cloned in pET 23a which is a T7 RNA polymerase inducible promoter based expression vector and were transformed in the BL21 (DE3) strain of *E. coli*. Isopropyl β-D-1-thiogalactopyranoside (IPTG) was used to induce protein expression. The bacterial pellet was disrupted by sonication. After centrifugation, the supernatant (soluble fraction) and the pellet (insoluble fraction) were analyzed using 12.5% polyacrylamide gel electrophoresis (SDS-PAGE) and stained with Coomassie Brilliant Blue. G-CSF was purified using the method reported earlier in Kumari et al. (2020). Briefly, the G-CSF protein was expressed as inclusion bodies, which were solubilized using 2 M urea. The solubilized G-CSF protein was subjected to refolding and purified using cation exchange chromatography (CEC).

### RT PCR for Different Constructs for Analyzing mRNA Expression Levels in *E. coli*

The RNA was extracted after IPTG induction using the RNeasy Kit from Qiagen (Cat No. 74104). The RNA extracted was also treated with RNase-Free-DNase from Qiagen (Cat. No. 79245). cDNA from different constructs were synthesized using the iScript^TM^ cDNA Synthesis Kit from Bio-Rad. Different primer sets covering full, 5′ and 3′ regions were used to check mRNA expression levels.

### Analytical Characterization of G-CSF

Purified G-CSF was analyzed using 15% SDS-PAGE and stained with Coomassie Brilliant Blue (CBB). Western blot analysis was performed to confirm the identity of the purified G-CSF protein. After running purified recombinant G-CSF protein and the commercially available product on sodium dodecyl sulfate-polyacrylamide gel electrophoresis (SDS-PAGE), the gel was electro-transferred onto the PVDF membrane. The membrane was blocked with 2% milk powder or 1% BSA in 1X PBS (phosphate-buffered saline) for 1–2 h. The blocking solution was removed, and the blots were incubated for 1 h with a monoclonal antibody (mAb) against human G-CSF at 1:1000 dilution in 1X PBS-T. The intact molecular mass of G-CSF and the control was determined using liquid chromatography-mass spectrometry (LC-MS) Agilent 6550 system. Circular dichroism (CD) spectrometry was performed, as mentioned earlier on the Jasco J-815 spectropolarimeter (Kumari et al., 2020).

### *In vitro* Biological Activity of G-CSF

The *in vitro* biological activity of G-CSF was determined by measurement of cell proliferation assay using the M-NFS-60 cell line (ATCC CRL- 1838). The cells were treated with various concentrations of WHO G-CSF standard and E8 G-CSF for 48 h and metabolic activity was assessed using standard XTT assay protocol. The cells were grown in RPMI-1640 medium containing 10% fetal bovine serum, 1X penicillin and streptomycin, 0.05 mM β-mercaptoethanol and 62 ng/ml of human recombinant macrophage colony-stimulating factor (M-CSF) as recommended by ATCC. M-NFS-60 cells (35,000 cells/well) were seeded into a 96-well flat-bottom plate containing 2% FBS and serum-starved for 24 h. Varying concentrations of GCSF and G-CSF standard (0.01, 0.1, 1, 10, 100, 1,000 pg/mL) were added to each well. G-CSF formulation buffer was used as a control. Each concentration was set up in triplicate. After 48 h of incubation, XTT was added to each well, and the cells were kept for incubation at 37°C for 4 h. The optical density of the 96 well plate was measured at 490 nm and at 650 nm to normalize the blank reading using an ELISA reader. Data were plotted using GraphPad Prism 6 for Windows, Version 6.05. The standard error of the mean (SEM) was calculated.

## Results

### Evaluation of Transcript Levels and Protein Expression From Native Human G-CSF and Codon-Optimized Variant of G-CSF

**FIGURE 1:**
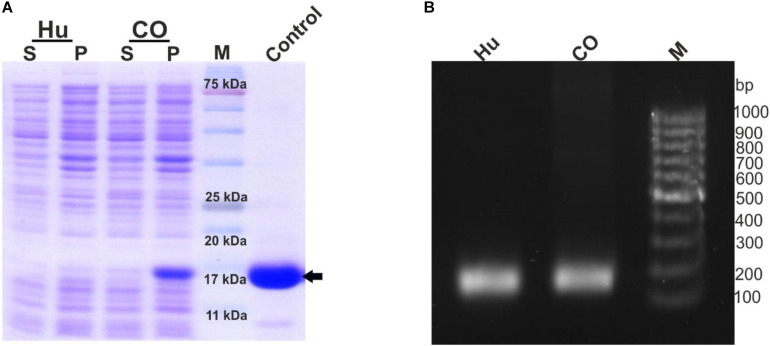
Low expression of G-CSF protein from constructs with native human cDNA and codon-optimized sequence despite sufficient transcription. **(A)** Depicts the protein expression profile from native human G-CSF and codon optimized G-CSF constructs. The constructs were overexpressed in *E. coli*. After sonication of IPTG induced transformed *E. coli*, the supernatant and pellet were analyzed on SDS-PAGE and stained with Coomassie Brilliant Blue. Hu indicates native human G-CSF, CO for codon optimized G-CSF. Control is commercially available G-CSF, which was used as positive control, the arrow head shows the G-CSF protein, S-supernatant, P-pellet and M-marker. **(B)** Depicts the RT-PCR profile of native human G-CSF and codon optimized G-CSF constructs. Primers (SD11 and SD2) targeting the 3′ end of the transcript were used for RT-PCR. The expected product size is 139bp.

This study was initiated to engineer a novel construct that could lead to an enhanced expression of the recombinant G-CSF in *E. coli*. Toward this, the cDNA sequence of human G-CSF was retrieved from NCBI GenBank using accession number M13008.1. This sequence was codon-optimized and synthesized (via GenScript, United States) for expression in *E. coli*. Both the native and the codon-optimized sequences were PCR amplified and cloned in pET-23a. The constructs were transformed in *E. coli* BL21 (DE3) strain to analyze the protein expression level. Protein expression from both the constructs was analyzed using SDS-PAGE analysis. It is important to note that the expression of recombinant G-CSF using native human sequence was extremely low, and even the codon-optimized sequence results in low expression (Figure 1A). Having seen low protein expression levels, mRNA levels of the constructs with native human G-CSF gene and codon-optimized gene were analyzed using RT-PCR analysis. This analysis suggested that transcripts from both the constructs are expressed sufficiently to comparable levels (Figure 1B).

### mRNA Structural Analysis and G-CSF Transcript Engineering

**FIGURE 2:**
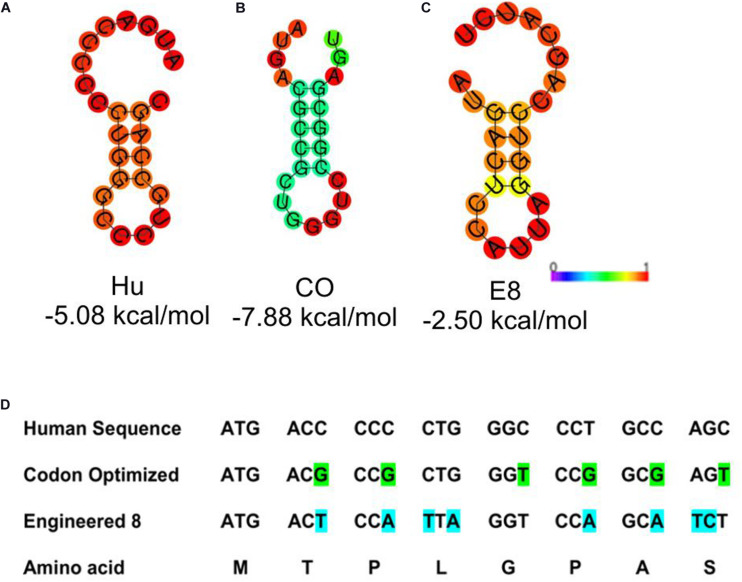
Depiction of stable secondary structure along with minimum free energy (MFE). **(A)** Native human G-CSF sequence; Hu **(B)** Codon optimized sequence; CO and **(C)** Engineered construct; E8. The free energy of the thermodynamic ensemble is also given below each drawing. The MFE structure is colored by base-pairing probabilities and for unpaired regions the color denotes the probability of being unpaired as depicted in the scale bar. The ViennaRNA Web Service (RNAfold web server) was used to predict the MFE structure. **(D)** Depicts the 5′ sequence alignment of native human G-CSF, codon optimized and engineered construct E8.

Stable secondary structures at the 5′ end of the transcript are known to modulate the mRNA’s translation efficiency. Thus we aimed at studying the presence of secondary structures at the 5′ end. To analyze the structural architecture of the 5′ region of the G-CSF transcript, computational analysis of the mRNA sequence was performed. RNAfold web server was used (Lorenz et al., 2011) to analyze the secondary structures at the 5′ end of the G-CSF transcript. This analysis suggested the formation of hairpins and highly stable secondary structures at the 5′ end of the mRNA transcript (Figures 2A–C), raising the possibility that these complex structures might be hindering the translation of the transcript. To further understand the secondary structures’ stability, the thermodynamic ensemble’s free energy was analyzed for the G-CSF transcript’s first 24 nucleotides. In agreement with the low expression of recombinant G-CSF, this analysis suggested a high GC content at 5′ prime end of the transcript and negative value of free energy, both of which predict the presence of highly structured stable RNA structures that could affect the translation efficiency (Table 2). These data indicate that the structural features of mRNA transcribed from the native G-CSF cDNA and its codon-optimized version may be hindering the translation efficiency.

**TABLE 2:** mRNA sequence of native, codon optimized and engineered G-CSF transcripts, their GC content and free energy of the thermodynamic ensemble.

Sequence ID	5′ G-CSF mRNA Sequence	GC content (%)	Free energy of the thermodynamic ensemble
Human Sequence HU	AUG ACC CCC CUG GGC CCU GCC AGC	75	−5.08 kcal/mol
Codon optimized CO	AUG ACG CCG CUG GGU CCG GCG AGU	70.8	−7.88 kcal/mol
Engineered E3	AUG ACU CCA CUG GGU CCG GCG AGU	62.5	−4.55 kcal/mol
Engineered E4	AUG ACU CCA UUA GGU CCG GCG AGU	54.2	−2.51 kcal/mol
Engineered E5	AUG ACU CCA UUA GGU CCA GCG AGU	50	−2.53 kcal/mol
Engineered E6	AUG ACU CCA UUA GGU CCG GCA AGU	50	−2.46 kcal/mol
Engineered E7	AUG ACU CCA UUA GGU CCA GCA AGU	45.8	−2.48 kcal/mol
Engineered E8	AUG ACU CCA UUA GGU CCA GCA UCU	45.8	−2.50 kcal/mol
Engineered E9	AUG ACU CCG CUG GGU CCG GCA UCU	62.5	−4.77 kcal/mol
Engineered E10	AUG ACU CCG UUA GGU CCG GCA UCU	54.2	−3.08 kcal/mol

*#Free energy of the thermodynamic ensemble was determined using ViennaRNA Web Service (RNAfold web server), this server predicts the secondary structure and minimum free energy of single stranded RNA.*

Next, we utilized the mRNA’s secondary structure and envisaged translationally silent mutations (Figure 2D) to disrupt the secondary structures. The mRNA encoded by these sequences have free energy of the thermodynamic ensemble in a range of −2.46 kcal/mol to −7.88 kcal/mol, and GC content range from 45 to 75% (Table 2). Furthermore, the analysis of secondary structures of designed sequences using the RNAfold web server suggested that the mutagenesis would lead to instability in the secondary structures in the 5′ of the G-CSF transcript and decrease the base-paring probability. The free energy of the thermodynamic ensemble of the highly structured RNA was more negative, and it was reduced upon the engineering of the G-CSF transcript (Figure 2).

### G-CSF Protein Expression and Purification

**FIGURE 3:**
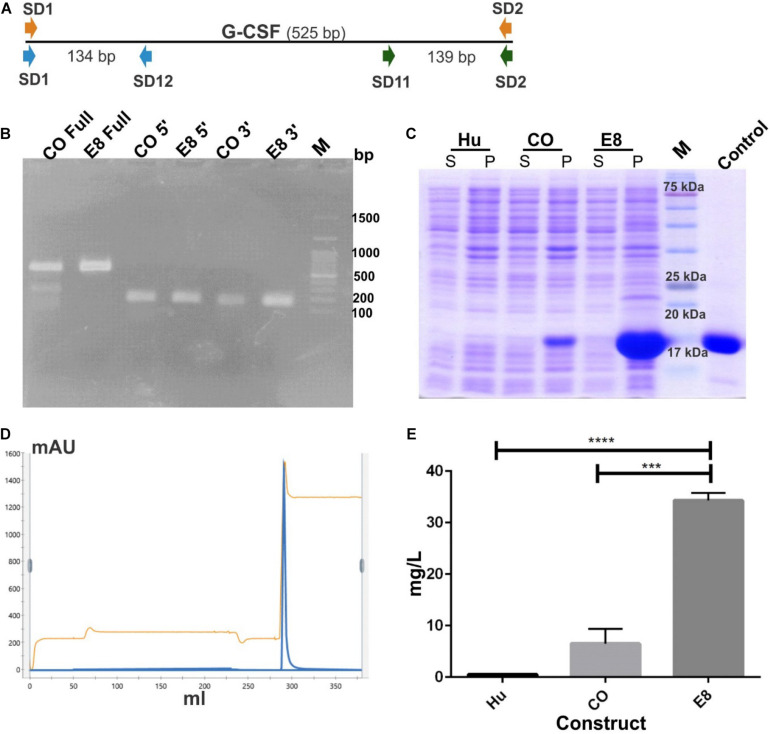
Increase in protein expression of G-CSF through mRNA engineering without affecting transcription. **(A)** Depicts the position of different primer sets used in RT PCR. Three different primer sets were used covering full length of the G-CSF transcript; Full (SD1 & SD2 for CO and SD8 & SD2 for E8), 5′ prime end; 5′ (SD1 & SD12 for CO and SD8 & SD12 for E8) and 3′ prime end; 3′ (SD11 & SD2 for both CO and E8) of the transcript. **(B)** Depicts the RT-PCR profile of G-CSF codon-optimized (CO) and engineered construct (E8). Expected RT-PCR product size is 525, 134, and 139 bp respectively. **(C)** Depicts the protein expression profile of native G-CSF, codon-optimized G-CSF and engineered G-CSF construct, E8. The constructs were overexpressed in *E. coli*. After sonication of IPTG induced *E. coli*, the supernatant and pellet were analyzed on SDS-PAGE and stained with Coomassie Brilliant Blue. Lane identity are given at top; Hu-Native human cDNA sequence, CO-codon optimized G-CSF, E8-engineered G-CSF construct. Commercially available G-CSF was used as control. M- marker, S- supernatant and P- pellet. **(D)** Cation exchange chromatography profile of the G-CSF. Protein was eluted by using 1 M Tris-Cl. Parameters such as absorbance at 280 nm and conductance have been represented with blue and brown lines, respectively. **(E)** Bar graph depicting the yield of CEC purified G-CSF from construct using native human cDNA sequence (Hu), construct using codon-optimized gene for G-CSF expression (CO) and engineered construct (E8). Data are Mean ± SEM from three biological experiments. Students’ *t-*test was performed for establishing the statistical significance of the results. ****indicates a *p* value of <0.0001 and *** indicates *p* value of 0.001.

We selected the engineered 8 variant (referred to as E8 in this manuscript) with eight translationally silent modifications for further analyses (Figure 2D). The translationally silent mutations were introduced using PCR primers with the desired substitutions at the specific positions in the 5′ coding sequence. This engineered construct was transformed in BL21 (DE3) strain of *E. coli* to analyze protein expression levels. To investigate the effect of these translationally silent mutations on G-CSF transcription, RT-PCR analysis was performed. We utilized different primer set targeting the full-length transcript, or the 5′ end or the 3′ end of the transcript (Figure 3A). No significant difference in the transcript expression of the G-CSF transcript between the engineered construct and the codon-optimized construct was observed (Figure 3B).

The E8 variant, along with the native G-CSF and codon-optimized constructs, were compared for the protein expression levels to determine the effect of translationally silent mutations on the translational efficiency. Interestingly, a substantial increase in the protein expression from the modified transcript was observed. The enhanced G-CSF protein expression was observed in the E8 variant (Figure 3C). To quantitate the increase in translational efficiency, G-CSF was expressed from these constructs, followed by protein purification and quantitation of purified protein. The G-CSF protein resided in the inclusion bodies and was purified using urea followed by two-step refolding and cation exchange chromatography (Figure 3D). The purified protein was quantitated using Thermo Scientific NanoDrop 2000. Interestingly, engineered construct E8 showed a 30–60 fold increase in CEC purified protein as compared to the native human G-CSF sequence (Figure 3E).

### Physicochemical Characterization of E8 G-CSF

**FIGURE 4:**
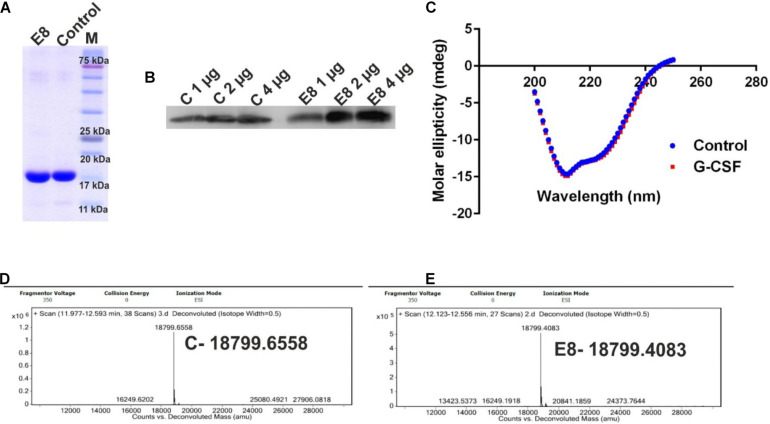
Characterization of the E8 G-CSF variant. **(A)** Depicts the SDS-PAGE profile of eluted E8 G-CSF protein and the commercially available G-CSF as control, exhibiting the general purity of the eluted G-CSF protein. **(B)** Shows the western blot analysis of G-CSF proteins 1, 2, and 4 μg of protein sample were used. **(C)** Shows a comparison of far UV circular dichroic spectra of G-CSF and commercial available G-CSF. **(D)** Depicts the LC/MS prolife of control protein. **(E)** Depicts the LC/MS prolife of E8 G-CSF protein. Control/C stands for commercial available G-CSF protein.

The purified protein was analyzed using SDS-PAGE (Figure 4A). Western blot analysis was performed to assess the quality and identity of G-CSF protein (Figure 4B). Commercially available G-CSF as control was also included in these gels, and data from both the analyses confirmed the purity and identity of purified G-CSF protein from E8 construct. Circular dichroism (CD) spectroscopy was performed for probing the secondary structure of G-CSF and control. G-CSF possesses a predominantly helical structure, wherein four-alpha-helices are connected through unstructured regions. We observed that the far-UV CD spectra of E8 G-CSF and control G-CSF were practically identical (Figure 4C). The spectra had the same shape, indicating alpha-helices’ predominance in both the proteins. The superimposed spectra from control G-CSF and the E8 G-CSF is suggestive of identical secondary structures. The intact molecular mass of E8 G-CSF and the control G-CSF was determined using liquid chromatography-mass spectrometry (LC-MS) and was found to be similar (Figures 4D,E).

### *In vitro* Biological Activity of G-CSF

**FIGURE 5:**
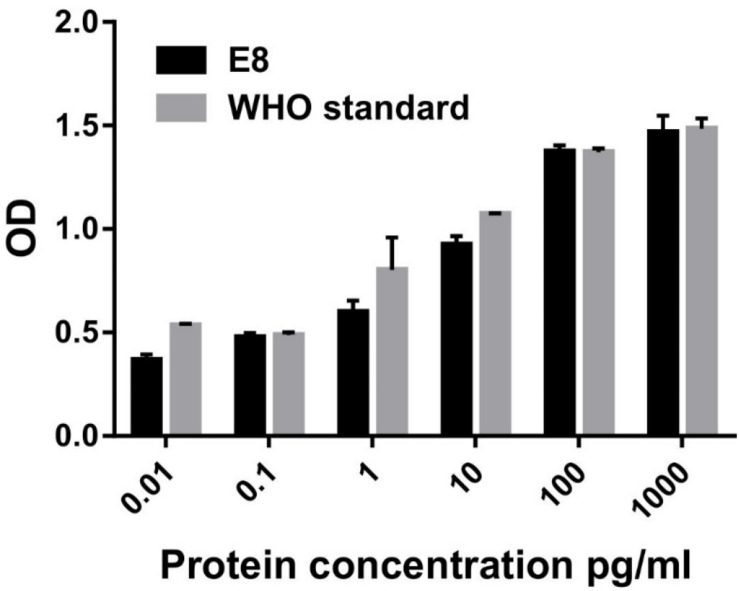
*In vitro* biological activity of G-CSF using M-NFS-60 cell line. 0.035 million M-NFS-60 cells were seeded into a 96-well flat-bottom plate containing 2% FBS and serum-starved for 24 h. Varying concentrations of GCSF and G-CSF standard (0.01, 0.1, 1, 10, 100, 1,000 pg/mL) were added to each well. 48 h after providing the cytokine, cellular proliferation was analyzed using XTT assay. G-CSF WHO standard was used as a positive control. Data are Mean ± SEM.

Next, we also analyzed the *in vitro* biological activity of G-CSF protein using a G-CSF responsive M-NFS-60 cell line. The treatment of M-NFS-60 cells to increasing concentration of G-CSF induces a dose-dependent proliferation of M-NFS-60 cells. In this study, the WHO G-CSF standard obtained from National Institute for Biological Standards and Control (NIBSC) was used as a reference. The results demonstrate that E8 possesses similar cell proliferative activity/potential as that of the G-CSF standard (Figure 5). Thus, the results highlight that the free energy value of the first 24 nucleotide thermodynamic ensemble is crucial in predicting translational efficiency. This study suggested that such an approach could also be employed to increase the expression of other therapeutic proteins, wherein even mRNA engineering of the first 24 nucleotides in transcript could result in a substantial increase in protein expression. These findings delineate a simplified and robust strategy that could increase the protein expression of other therapeutic proteins and proteins required for research applications.

## Discussion

G-CSF is an important biotherapeutic protein. Given its market value, it needs to be produced in large quantities. In this study, it was observed that native human cDNA sequence for G-CSF and its codon-optimized variant are transcribed with reasonable efficiency in *E. coli* through T7 promoter. However, the protein expression for both the two transcripts was low. Analysis of the secondary structures in the 5′ end of the transcript revealed the presence of stable secondary structures with high base pairing probability. Disruption of stable secondary structures at the 5′ end of the transcript resulted in a significant increase in the protein expression. Thus, this study has outlined a strategy for optimizing transcript sequence to improve the protein expression of the clinically/industrially important proteins.

High expression of therapeutic proteins is always desired. Several studies have earlier utilized different methodologies for improving the yield of G-CSF protein. Most of the studies have focused on modification of media composition, IPTG use (Vanz et al., 2008; Boubeva et al., 2012), ethanol utilization (Mishra et al., 2020), optimization of fermentation (Kim et al., 2014), or subsequent purification strategy/methodology (Wang et al., 2008; Li et al., 2012; Vemula et al., 2015) for improving the yields of G-CSF protein. Codon optimization of G-CSF for improved expression has been extensively used for expression in yeast (Maity et al., 2016) and *E. coli* (Gomes et al., 2012). Literature also suggests that the optimization of AT-content of codons immediately downstream of the initiation codon for high-level G-CSF expression. The culture conditions were also optimized wherein 1% glucose was used as a supplement, and *E. coli* BL21 (DE3) PLysS strain was utilized. The productivity was increased 1.5 folds (Krishna Rao et al., 2008).

However, the genetic architecture of the gene encoding G-CSF or its transcript has been largely ignored. Importantly, mRNA secondary structure regulates protein expression by modulating the transcript’s functional half-life (Mauger et al., 2019). Literature is indicative of an important role of mRNA secondary structure optimization in modulating translation efficiency and thus the protein expression. One of the classical studies by Kozak delineated the influence of mRNA secondary structures on the initiation of translation by eukaryotic ribosomes. In this study, the incorporation of artificial secondary structures into the 5′ non-coding region of a chimeric mRNA encoding preproinsulin suggested that the 40s ribosomal subunits could melt hairpin structures with Gibbs energy in the range of −30kcal/mol, but secondary structures in the range of −50 kcal/mol resist melting and inhibit translation up to 85–95% (Kozak, 1984, 1986).

In the light of this literature, this study was initiated with the objective of engineering G-CSF transcript for enhanced recombinant G-CSF expression in *E. coli*. It was observed that the native human and *E. coli* codon-optimized sequences lead to low to moderate expression of G-CSF. Thus, a computational analysis of the 5′ end of G-CSF transcript from the native human sequence and the codon-optimized gene was carried out. We specifically focused on the RNA structure of the first 24 nucleotides rather than the structural prediction of entire mRNA since secondary structures in the 5′ end of the transcript play a more prominent role in regulating translation initiation. This analysis revealed the presence of stable secondary structures at the 5′ end of the transcript. These stable structures were driven by higher GC content at the 5′ end of the transcript. We utilized computational biology for rational sequence designing of several 5′ sequences to disrupt the stable secondary sequences to enable efficient translation initiation from the transcript. Toward this, we utilized analysis of GC content and free energy levels. The codon-optimized transcript was modified using translationally silent mutations. These translationally silent mutations were explicitly aimed at reducing the mRNA secondary structure complexity by replacing critical GC-rich regions with AT-rich regions. The percentage codon usage suggesting the frequency of the codon used per 100 codon information was also considered while incorporating changes. Emphasis was given on reducing the transcript structural complexity by decreasing the base pair probability in the paired region and increasing the probability of being unpaired in the unpaired region of transcript structure, irrespective of the Codon Adaptation Index (CAI) of the modified sequence (Sharp and Li, 1987). The rationale for modifying the 5′ end of the transcript was that the RNA secondary structures after initiation codon could act as a roadblock to impact the ribosome scanning during translation and impact translational efficiency negatively. The physicochemical characterization and immunoassay suggested that the G-CSF protein resulting through modification of transcript was identical to the commercially available G-CSF. Furthermore, *in vitro* biological activity was also studied and was comparable with WHO standard of G-CSF protein. Thus the transcript engineering involving translational silent mutations results in enhancing the protein expression and biologically active protein.

This study has suggested that the protein expression from the construct having native human cDNA for G-CSF and the codon-optimized gene for recombinant G-CSF is low to moderate due to the formation of stable secondary structures at the 5′ end of the G-CSF transcript. Translationally silent mutagenesis within the first 24 bp in the G-CSF construct could dramatically disrupt the stable secondary structures to increase translational efficiency and G-CSF yield. This study suggests that such an approach could also be employed to increase the yield of other therapeutic proteins, wherein even engineering of the first few nucleotides in the transcript could result in a substantial increase in protein yield.

## Conclusion

Thus study suggests that the protein yield from the construct with native human cDNA G-CSF and codon-optimized sequence for recombinant G-CSF is low due to stable secondary structures at the 5′ end of the transcript. Translationally silent mutagenesis within the first 24 nucleotides of the G-CSF construct was employed to disrupt the stable secondary structures. This led to a dramatic increase in translational efficiency and enhanced protein expression. Interestingly, the free energy value of the first 24 bp thermodynamic ensemble is crucial in predicting translational efficiency. This study suggested that such an approach could also be employed to increase the yield of other therapeutic proteins and proteins involved for research purposes. Importantly, even engineering of the first few nucleotides in the transcript could substantially increase protein yield.

## Data Availability Statement

The original contributions presented in the study are included in the article/supplementary material, further inquiries can be directed to the corresponding author/s.

## Author Contributions

SD conceived the idea, performed the experiments, interpreted the data, and wrote the manuscript.

## Conflict of Interest

The author declares that the research was conducted in the absence of any commercial or financial relationships that could be construed as a potential conflict of interest.
